# Lesion-based analysis of ^18^F-FDG uptake and ^111^In-Pentetreotide uptake by neuroendocrine tumors

**DOI:** 10.1007/s12149-014-0900-3

**Published:** 2014-09-02

**Authors:** Kazuo Kubota, Momoko Okasaki, Ryogo Minamimoto, Yoko Miyata, Miyako Morooka, Kazuhiko Nakajima, Takashi Sato

**Affiliations:** Division of Nuclear Medicine, Department of Radiology, National Center for Global Health and Medicine, 1-21-1 Toyama, Shinjuku-ku, Tokyo, 162-8655 Japan

**Keywords:** 18F-fluorodeoxyglucose, FDG, Positron Emission Tomography (PET), ^111^In-pentetreotide, Somatostatin receptor scintigraphy, Neuroendocrine tumor, Tumor heterogeneity

## Abstract

**Purpose:**

To characterize the heterogeneity of metastatic neuroendocrine tumor (NET) lesions, we compared the [^18^F]-fluorodeoxyglucose (FDG) uptake and the ^111^In-pentetreotide (SRS) uptake for somatostatin receptor scintigraphy using the CT-based fusion imaging techniques of PET/CT and SPECT/CT.

**Methods:**

Fifteen consecutive patients with NET lesions were examined using both FDG-PET/CT and SRS SPECT/CT prospectively. A total of 45 metastatic NET lesions were evaluated for FDG uptake according to the standardized uptake value (SUV) and for SRS uptake according to the tumor-to-muscle count ratio (T/M ratio); these values were then compared according to the grade of NET (G), also compared to the tumor volume.

**Results:**

Both the SRS uptake and FDG uptake showed no significant correlation to the tumor volume, and suggested no significant artifacts in these data. The T/M ratio for the SRS uptake ranged from 192.7 to 1.9 and exhibited very wide range of distribution. The SUV for the FDG uptake ranged from 13.8 to 0.77 and exhibited narrow range of distribution. The uptake of the two tracers in individual lesions showed an inverse correlation. The G1 + 2 lesions had a higher SRS uptake than the G3 lesions, but the difference was not significant because of the large variation (40.65 ± 48.03, *n* = 39 vs. 8.66 ± 13.13, *n* = 6). However, the G1 + 2 lesions had a significantly lower FDG uptake than the G3 lesions (3.52 ± 1.84, *n* = 39 vs. 10.82 ± 4.50, *n* = 6). The tracer uptakes varied largely not only in an inter-subject manner, but also in an intra-subject manner.

**Conclusion:**

An inverse correlation between SRS uptake and FDG uptake in the metastatic NET lesions observed in this study may be consistent with the opposing ideas of differentiation and proliferation in oncology. The large variations in SRS and FDG uptake by metastatic NET lesions suggest the biological heterogeneity of advanced NET. These results support the idea that combination therapy targeting both receptor-positive cells and proliferating cells may be beneficial from a functional imaging perspective.

## Introduction

Neuroendocrine tumors (NETs) arise from the neuroendocrine system and are characterized by their biological diversity, such as functioning or non-functioning and rapid growth or stable course [1, 2]. Because of the diverse nature of this disease and its relatively rare prevalence, the management of patients with NET is sometimes difficult. Some physicians have proposed a policy of “watch-and-wait” until tumor progression for patients with well-differentiated tumors. However, such a strategy can lead to the potentially curative period being missed. NETs are usually asymptomatic during their early stages and are often discovered because of symptoms arising from metastatic spread. The early recognition of a tumor’s potential for progression seems to be important.

Somatostatin receptor scintigraphy using ^111^In-pentetreotide (SRS; Octreoscan^®^) is a standard technique for the diagnosis of neuroendocrine tumors (NET) and is recommended by treatment guidelines [3]. Recently, the possibility of using FDG-PET to evaluate the aggressiveness of NET has been reported. NET patients with a low FDG uptake reportedly had a better outcome than those with a higher FDG uptake, while patients with a high SRS uptake reportedly showed a better outcome than those with a lower SRS uptake [4]. FDG uptake might reflect the grade of malignancy, i.e., proliferation, while SRS uptake might reflect the grade of differentiation. This hypothesis is consistent with the different roles of FDG-PET and SRS in the evaluation of NET. However, lesion-based analyses of individual patients with metastatic NET have not yet been reported.

To characterize the heterogeneity of metastatic NET lesions, we compared the FDG uptake and the SRS uptake of individual metastatic NET lesions using the CT-based quantitative imaging techniques of PET/CT and SPECT/CT.

## Subjects and methods

### Subjects

Fifteen consecutive patients (10 female, 5 male, mean age 58.4 years, range 44–72 years) with neuroendocrine tumors (NETs) were prospectively enrolled in this study between September 2011 and July 2013. Fourteen patients were evaluated for metastatic NET disease after undergoing surgery, and one patient was evaluated prior to undergoing surgery. Any long-acting octreotide analog therapy had been suspended for 4 weeks prior to enrolment in the study. The 2010 WHO grades (G) of NET were as follows: G1, 2 patients; G2, 11 patients; G3, 2 patients. The patient characteristics are shown in Table 1. Written informed consent was obtained from all the patients, and the study protocol was approved by the Internal Review Board of our institution.

**Table 1:** Data table of neuroendocrine tumor patients (10 females, 5 males. Mean age, 58.4 years (range, 44–72 years).)

NET grade	No. patients	No. lesions	Primary site (no. patients)
Grade 1	2	1	Stomach (1), Pancreas (1)
Grade 2	11	38	Thymus (2), Pancreas (4), Small Intestine (2) Duodenum (1), Kidney (1), Unknown primary (1)
Grade 3	2	6	Lung (2)
Total	15	45	

### Imaging methods

An ^111^In-pentetreotide labeling kit (OctreoScan; Mallinckrodt, Petten, The Netherlands) was purchased for personal use with a certificate for the import of medicines, as this kit has not yet been approved for use in Japan. The ^111^In-labeling and the quality control were performed in our department according to the manufacturer’s recommendations. The radiochemical purity was examined using a thin-layer chromatography (TLC) method, and a purity of over 95 % was considered appropriate for injection. A dose of 111–222 MBq of ^111^In-pentetreotide (SRS) was intravenously injected. Whole body anterior and posterior planar images and several spot images were obtained at 4, 6, and 24 h after injection. SPECT/CT imaging with attenuation correction of the abdomen and pelvis as well as the chest, if necessary, was performed at 6 and 24 h after injection for all the patients. For three patients, additional SPECT/CT images were obtained at 4 h after injection. In one patient, additional imaging at 48 h after injection was performed. The bowel excretion was seen at 24 h, but laxative therapy was not performed in this study. A dual-headed gamma camera equipped with a medium-energy, general-purpose, parallel-hole collimator and a low-dose CT unit (VG Hawkeye; GE Healthcare) was used. SPECT was performed using 6 steps, 40 s/step, a 180° orbit, and a matrix size of 128 × 128. The low-dose CT images obtained simultaneously were used for both attenuation correction and the anatomic localization of the SPECT images using a dedicated workstation (AW; GE Healthcare).

The standard imaging times for SRS are at 4, 24, and 48 h after injection, according to diagnostic guidelines [5]. Because of scheduling problems in performing SPECT/CT, we performed the imaging studies at 6 and 24 h after injection in all the patients. To confirm the effect of the imaging time on the T/M ratio and the actual tumor counts, we analyzed the time activity curves for the tumor, liver and muscle in 3 patients (Grade 2 disease; total of 14 metastases) using SPECT/CT data obtained at 4, 6, and 24 h after injection.

[^18^F]-fluorodeoxyglucose (FDG) was synthesized using an in-house cyclotron and automated synthesis system (F200; Sumitomo Heavy Industry) using the authorized procedure. After fasting for 6 h or more, the blood glucose level of each patient was measured and the patient was intravenously injected with 5 MBq/kg of FDG. One hour after FDG injection, PET/CT imaging from the vertex to the toe (with the arms in a downward position to enable a comparison with the SRS images) was performed using a dedicated PET/CT scanner (either a Biograph 16 [Siemens] or a Discovery PET/CT 600 [GE Healthcare]) with a 2-min emission scan/bed and CT attenuation correction. Low-dose CT was performed first and was used for attenuation correction and image fusion. The PET data were reconstructed using a Gaussian filter with an ordered-subset expectation maximization algorithm. The iteration and subset parameters were determined according to the manufacturer’s recommendations. A quality control study was performed to minimize the SUV difference between the two PET/CT systems using a standard NEMA body phantom.

The FDG-PET/CT study was performed the day before the SRS study in all the patients, and all the imaging studies were completed within 4 days.

### Analysis methods

Tumor lesions with either focal FDG uptake or SRS uptake and that were confirmed as mass lesions based on CT images were analyzed. The same lesion was carefully identified on both the FDG-PET/CT and SRS-SPECT/CT images using a side-by-side comparison on the same workstation (Xeleris; GE Healthcare). Up to 5 lesions per patient were analyzed to avoid the patient-based bias. FDG uptake and SRS uptake in the same lesion were evaluated as the SUVmax for the FDG uptake and the count ratio of the tumor-to-muscle (T/M) ratio for the SRS uptake by drawing a region of interest (ROI) that encompassed each tumor lesion. The average of three ROIs with diameters of 2–3 cm placed on the shoulder muscle or gluteus muscle were used as the mean muscle activity. The consistency of SRS activity in two muscle areas was confirmed separately. The maximum tumor count was used to calculate the T/M ratio.

Size of all the lesions was recorded and evaluated to see if size of the lesions affects the measured activity of tumor uptake of each tracer. We have used the Tumor Volume as the indicator of tumor size which is defined as the product of long axis (cm) and short axis (cm) of the tumor lesions. The measurements were performed using the contrast-enhanced CT obtained within 1 month interval of the SRS and FDG-PET/CT study. The smallest size of the tumor was 1.1 × 1.1 cm.

Note that the SRS-SPECT/CT images were attenuation-corrected but were not corrected according to the injected dose or the patient’s body weight. To compensate these variables, we used T/M ratio. Non-tumor lesions, such as granulomas, at the sites of intra-muscular injections of long-acting octreotide were carefully avoided.

### Statistical analysis

Mann–Whitney’s *U* test was used to examine the statistical differences of two data groups where normal distribution was not confirmed.

## Results

The SRS uptake by some of the tumors became highest at 6 h, and the rest of tumors at 4 h, and then decreased to that at 24 h. All the lesions identified at 6 h were also visible at 24 h, and none of the lesions were only seen at 24 h. Subsequently, we analyzed the data obtained at 6 h for all the patients (see the figure in the “Appendix” and discussion).

A patient with a G1 NET who showed no sign of a lesion after surgery and who had not developed a recurrence at a 1-year follow-up examination was excluded from the analysis. Consequently, a total of 45 lesions in 14 patients (1 lesion in a patient with G1 disease, 38 lesions in 11 patients with G2 disease, and 6 lesions in 2 patients with G3 disease) were analyzed (Table 1).

**Fig. 1:**
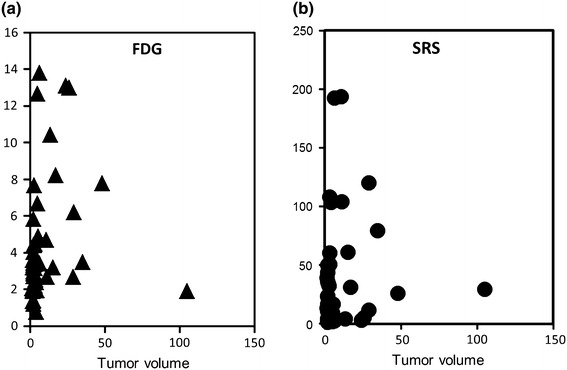
**a** (*left*) FDG uptake (SUV) by tumor lesion was plotted against tumor volume. **b** (*right*) SRS uptake (T/M ratio) by tumor lesion was plotted against tumor volume

FDG uptake (SUV) by tumor lesion was plotted against Tumor Volume (Fig. 1a). Also, SRS uptake (T/M ratio) by tumor lesion was plotted against Tumor Volume (Fig. 1b). The Fig. 1a clearly showed that FDG uptake by tumors showed no significant correlation to the tumor volume. The Fig. 1b also showed that SRS uptake showed no significant correlation to the tumor volume. Then, we can estimate that FDG uptake measured by PET/CT as SUV, SRS uptake measured by SPECT/CT as T/M ratio seem to represent the biological characteristics of each NET lesion.

**Fig. 2:**
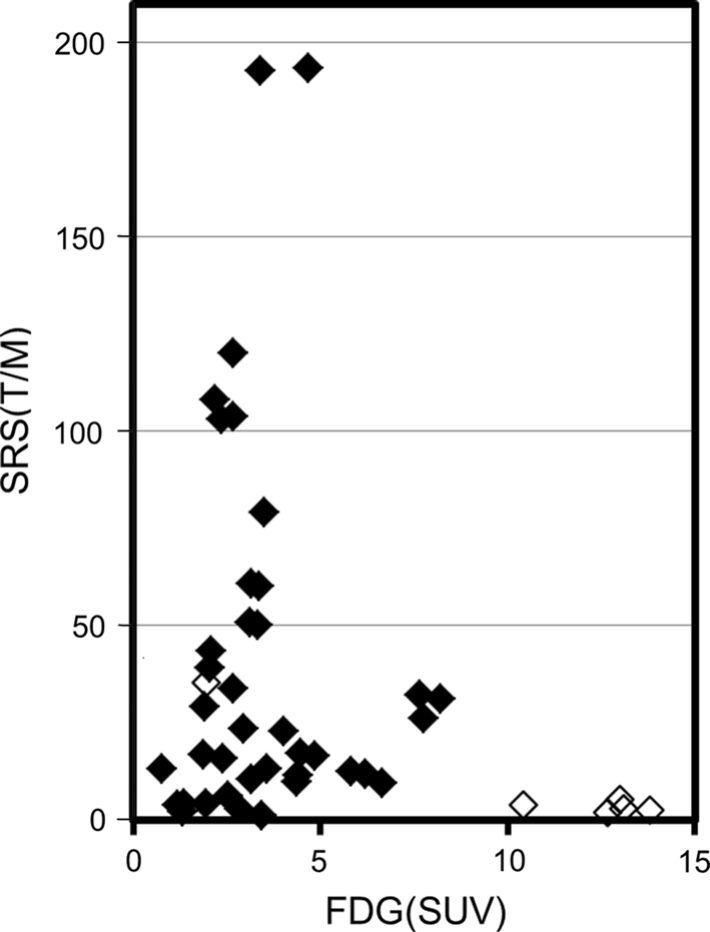
Correlation between FDG uptake (SUV) and SRS uptake (T/M ratio) by individual NET lesions. The uptake of the two tracers in individual lesions showed an inverse correlation. Solid symbol is G1 + 2 lesion, open G3 lesion

The mean and SD of the FDG and SRS uptake values in the G1 + 2 NET lesions and the G3 NET lesions are shown in Table 2. FDG uptake was significantly higher in the G3 lesions than in the G1 + 2 lesions (*P* < 0.01, Mann–Whitney’s *U* test). SRS uptake was higher in the Grade 1 + 2 lesions than in the G3 lesions, but the difference was not statistically significant because of the large variations. The mean, SD, and median of the tumor volume was also presented in Table 2. There are no significant differences in these tumor volume data of Grade 1 + 2 vs Grade 3 by Mann–Whitney’s *U* test. It suggested that the data of FDG uptake and SRS uptake presented in the Table 2 were not affected by the difference of the tumor volume but reflected only by the Grade of the tumors.

**Table 2:** WHO tumor grade and tracers uptakes (Mean ± SD, (Median))

WHO grade (*n*)	Grade 1 + 2 (*n* = 39)	Grade 3 (*n* = 6)
FDG (SUV)	3.52 ± 1.84 (3.17)	10.82 ± 4.50* (12.8)
SRS (T/M)	40.65 ± 48.03** (22.87)	8.66 ± 13.13 (3.4)
Tumor volume^a^	9.81 ± 8.83 (3)	12.67 ± 10.22 (9.78)

Mann–Whitney’s *U* test *P = 0.0045, **P = 0.0106

^a ^Tumor volume was the product of long axis (cm) and short axia (cm)

The correlation between the FDG uptake and the SRS uptake in individual NET lesions is shown in Fig. 2. Lesions with a higher SRS uptake had a variable, but generally lower, FDG uptake. Meanwhile, the lesions with a high FDG uptake had a lower SRS uptake. The T/M ratio of the SRS uptake ranged from 192.7 to 1.9 and had very wide range of distribution. The SUV of the FDG uptake ranged from 13.8 to 0.77 and had relatively narrow distribution.

**Fig. 3:**
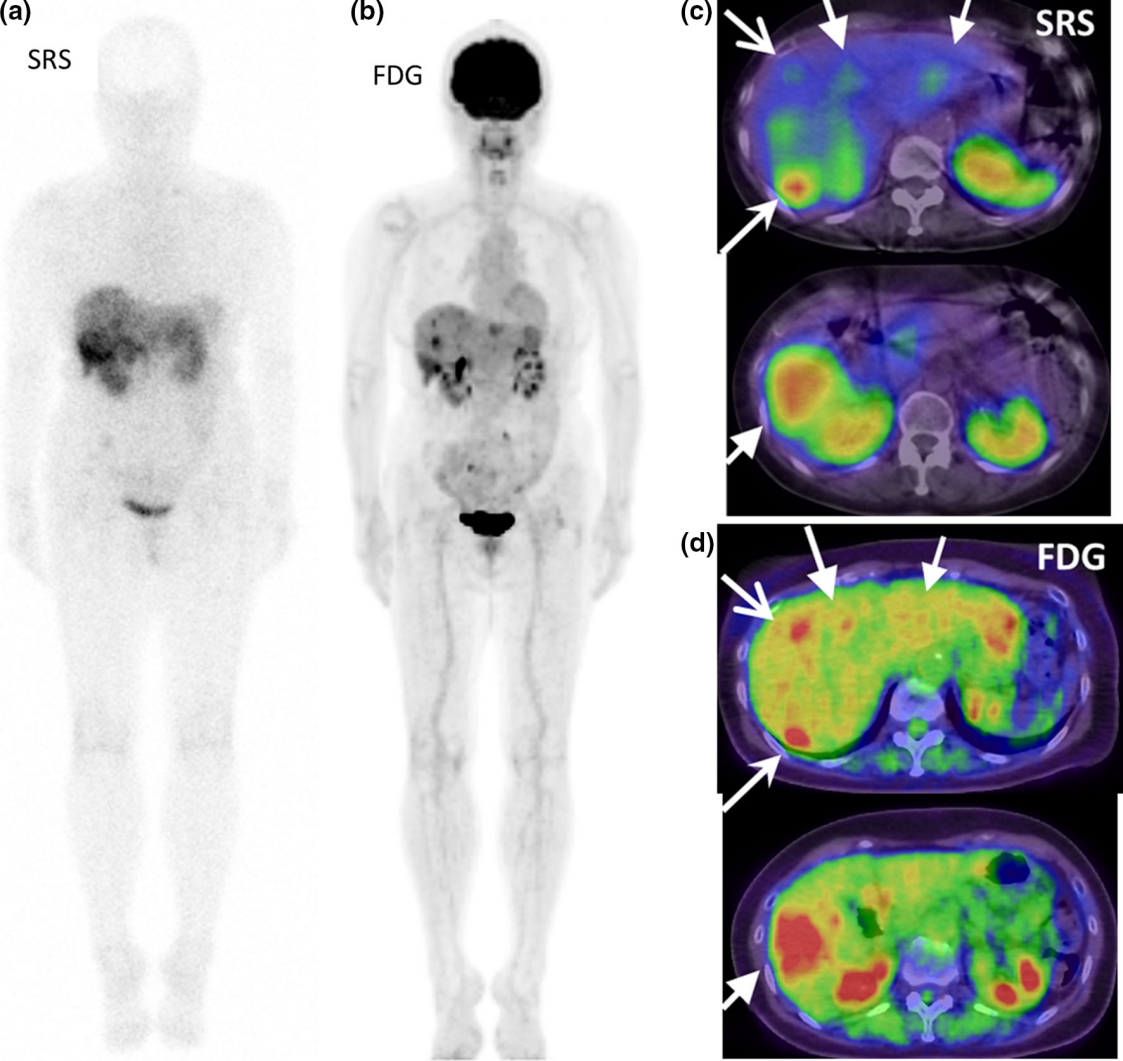
FDG-PET/CT images (**b**, **d**) and SRS images (**a**, **c**) of multiple liver metastases of G2 NET, unknown primary. Wholebody images (**a**, **b**) and two sets of axial images (**c**, **d**). Metastatic lesions in liver showed very large intra-patient variations in tracer uptake. Each arrow set in **c** and **d** indicated the same lesion seen in SRS and in FDG. Right posterior tumor showed high uptake both in FDG and in SRS, but right anterior tumor showed high uptake in FDG but low uptake in SRS

Typical images obtained for a G2 NET patient are presented in Fig. 3. Metastatic lesions in lever showed very large intra-patient variations in tracer uptake.

## Discussion

In this study, we evaluated SRS uptake and FDG uptake by individual metastatic NET lesions using quantitative imaging with SPECT/CT and PET/CT. We found that G1 + 2 lesions had a higher SRS uptake than G3 lesions, although the difference was not significant because of the large variations in uptake. However, the G3 lesions had a significantly higher FDG uptake than the G1 + 2 lesions. Lesion-based comparisons of SRS uptake and FDG uptake showed an inverse correlation, as well as large variations in the uptakes of both tracers by each metastatic lesion, even those within the same patient.

We analyzed the SRS data obtained at 6 h for all the patients. SRS uptake contrast at 24 h seems to be strongly affected by the clearance rate from 6 to 24 h (see figure in the “Appendix”). Ex vivo or in vivo experiments of somatostatin receptor binding of ^111^In-octreotide suggested specific binding of ligand may complete early after injection such as half hour in small animal model [6, 7]. In human, blood clearance is much slower and tissue delivery may continue 2–3 h [8]. The SRS uptake by tumor may reach its peak at several hours after, such as 4 or 6 h. It is well-established that evaluation of the receptor density in PET imaging should be performed at the equilibrium state between the in-put and the tissue concentration of the tracer. So, the early uptake of SRS by tumor seems to show the receptor density more accurately than the late uptake that will be much influenced by the clearance.

There are several arguments on the accuracy of SPECT/CT. Factors including, lesion size, respiratory movements may affect the accuracy for quantification of tracer uptake both using SPECT/CT or PET/CT. We examined the accuracy of quantification with phantom experiments, and found that small object less than 22 mm in diameter may be underestimated even in PET/CT, in SPECT/CT it may be worse (Data not shown). However, the most important point is the magnitude of the artifact if it has distorted the results or not. To examine this point, we have checked the correlation of the actual tumor volume and the measured activity of both tracers. Figure 1a and b, also Table 2 showed that both SRS uptake and FDG uptake showed no significant correlation to the tumor volume. So, our data seemed to show the characteristic of NET accurately.

Adams et al. reported the application of ^18^F-FDG-PET for NET as the only valuable predictor of malignancy in less-well-differentiated NET, and the use of FDG was recommended if SRS uptake was negative [9]. Belhocine et al. [10] reported that SRS was more useful than FDG for carcinoid tumors. The possible roles of SRS and FDG as prognostic indicators were reported by Garin et al. [4], they found that NET patients with a low FDG uptake had a better prognosis than those with a high FDG uptake, while patients with a high SRS uptake had a better prognosis than those with a low SRS uptake. They concluded that FDG-PET exhibited excellent predictive values for detecting early tumor progression. Also, Binderup et al. [11] reported a strong prognostic value of FDG-PET for NET that exceeded the prognostic value of conventional markers (Ki67, chromogranin A, and liver metastasis). Their comparisons of FDG-PET, SRS, and MIBG imaging in a large population of NET patients showed that the overall sensitivity of MIBG and FDG was lower than that of SRS. However, for tumors with a high proliferation rate, FDG-PET had the highest sensitivity [12]. So, FDG-PET is useful for revealing the aggressiveness of NET, while SRS is useful for revealing the expression of somatostatin receptors (in other words, the degree of differentiation). Our results that G1 + 2 lesions had a higher SRS uptake and that G3 lesions had a higher FDG uptake are consistent with previous reports and with the idea that proliferation and differentiation are opposing concepts in oncology.

The new WHO classification of 2010 is a nomenclature system that embraces the differentiation and grading features of NET beyond organ specificity, providing a valuable guide to the diversity of NET; this classification is both widely accepted and used [13]. However, the WHO classification is based on pathological criteria, such as the mitotic index, the MIB1 index, etc., which are determined using biopsied or resected specimens. While the pathological specimens that are obtained may be representative of the whole disease, there are no guarantees that all the metastatic/recurrent lesions will have the same characteristics. This study clearly showed the heterogeneity of such multiple metastatic lesions as large variations in SRS and FDG uptake even within the same patient.

The SRS uptake had a very wide range of distribution. While the FDG uptake had a relatively narrow distribution. Unique distribution pattern of SRS uptake may be reflected by the expression of somatostatin receptor. In vitro receptor assay of surgically removed NETs have reported that expression of somatostatin receptor in tumors showed very large variation that the differences of the highest and the lowest reached several hundred times [14]. It is quite different situation from the expression of glucose transporter 1(GLUT1) on the human tumor tissue that have been reported. These basic reports support our observation that SRS and FDG showed different distribution patterns.

Somatostatin receptor is a well-known therapeutic target for NET [15]. Long-acting octreotide and peptide receptor radionuclide therapy (PRRT) have been used widely. SRS uptake has been reported as a predictor of the response to PRRT [16]. The large variation in SRS uptake may suggest a limitation of somatostatin-based therapy against these multiple metastatic NETs. Recently, an inhibitor of mammalian target of rapamycin (mTor), everolimus, has been introduced as a new molecular targeting therapy for NET. The association between mTor expression and tumor growth is well-known [2]. Also, correlations between mTor expression and FDG uptake in mesothelioma [17] have been recently reported. If FDG uptake could become a surrogate marker for the therapeutic response of mTor inhibitor to NET, as reported in renal cell carcinoma [18], our data may suggest a limitation of mTor-based therapy against these multiple metastatic NETs. Finally, our data may support a combined SRS-based and proliferation-based therapy, such as the use of long-acting octreotide and an mTor inhibitor, because a combined therapy may better cover the heterogeneous characteristics of metastatic NETs. Bousquet et al. [19] reported that therapeutic trials combining somatostatin analogs and everolimus are a promising option based on a consideration of the molecular mechanisms of each agent. Our study may support this theory from the viewpoint of functional imaging. Further validation of this assumption is necessary.

## Conclusion

The inverse correlation between SRS uptake and FDG uptake in metastatic NET lesions observed in this study may be consistent with the opposing ideas of differentiation and proliferation in oncology. The large variations in SRS and FDG uptakes by metastatic NET lesions suggest the biological heterogeneity of advanced NETs. These results may support the idea that a combination therapy targeting both receptor-positive cells and proliferating cells may be beneficial from the viewpoint of functional imaging.

## Figures and Tables

**Fig. 4:**
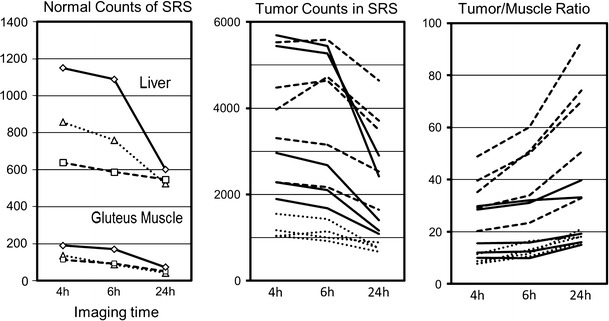
Time-activity curves of SRS uptake at 4, 6, and 24 h after injection in 3 patients with G3 NET. *Left* normal tissue counts of liver and gluteus muscle. *Middle* tumor counts of multiple metastatic lesions. *Right* tumor/muscle uptake ratio. Each line (*solid, dashed, dotted*) indicates the same patient. The T/M ratio increased significantly at 24 h because of the lower background. The rate of decrease in muscle uptake was larger than the decrease in tumor uptake, resulted in the elevation of tumor/muscle ratio at 24 h

## Appendix

See Appendix Fig. 4.
